# The Story of the Insane from Year to Year

**Published:** 1893-05-27

**Authors:** 


### THE STORY OF THE INSANE FROM YEAR TO YEAR.

### KENT COUNTY ASYLUM AT BARMING HEATH, NEAR MAIDSTONE.

On January 1st, 1891, there were 1,420 patients in this asylum, and on December 31st the number had risen to 1,536. Four hundred and seventy-five cases were admitted, 162 were discharged recovered, and 156 died. The recoveries come out at very close on 45 per cent. of the admissions, and the deaths stand at 10 3 on the average number resident. It would be useless to quote from the table showing the cause of insanity in those admitted. as nearly one-half are put down as "unknown," and 63 as "previous attacks." Twenty-seven of the deaths were owing to consumption. When referring to the Lunacy Acts Amendment Act of 1891, Dr. Davies says, "It was hoped this opportunity would be taken to modify some of the absurdities introduced so recklessly the year before. This is not so, however, and despite all the adverse criticism that remarkable Act has given rise to, the powers that be are content to go on blundering. Lunacy legislation is not a "party cry," it is difficult, therefore, to get any of our leaders interested in it. It has fallen into the hands of a set of faddists, who are ignorant of the needs of the lunatic, and too puffed up with intense belief in their own infallibility to seek advice."

### HULL BOROUGH ASYLUM.

In Table I. there would seem to be a misprint of "1890" for "1891," but assuming the numbers to refer to the latter year, we find there were in the asylum on the first day of the year 321 patients, and at the end of the year 324. There were 135 admissions, 43 recoveries, and 56 deaths. The percentage of recoveries on admission was 35·63, and the deaths on the average number resident, 17 per cent. It is a sad thing to chronicle that 38 of the patients admitted during the year owed their insanity to drink, and 31 to hereditary influence. Taken together we have these preventable causes accounting for over 50 per cent. of the year's insanity in the borough of Hull, and the ratepaying inhabitants may reckon what a nice little sum has to be spent annually, and the philanthropist may moralise on the amount of misery which has ensued, until surely both will come to the conclusion that it is time something was done to put an end or at least lessen these causes of insanity. 33 of the deaths were due to brain diseases, and 9 to pulmonary consumption. Dysenteric diarrhœa appeared in this asylum, and caused death in two cases. Like so many of his fellow superintendents, Dr. Merson could not trace this disease to its origin. The weekly charge is 11s. 8d. per head per week, and private patients are admitted at rates varying from 15s. to 28s. a week.

